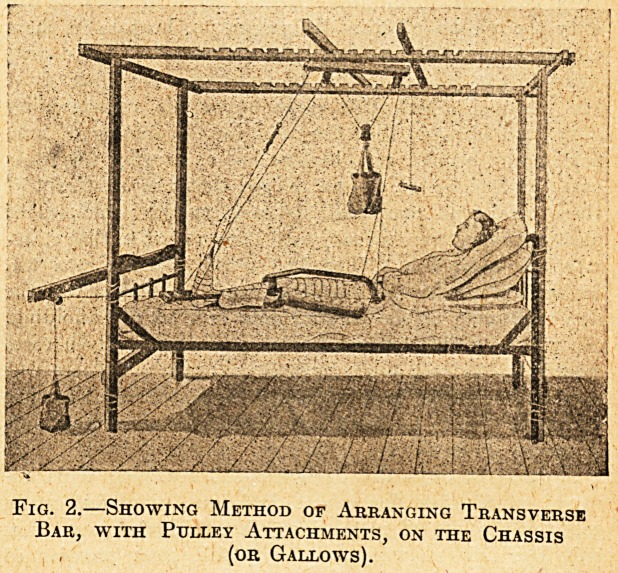# Hammock Suspension of Fractures

**Published:** 1917-12-08

**Authors:** 


### NEW APPLIANCES.

### Hammock Suspension of Fractures.

Possibly in no branch of surgery has more genius been brought to bear during the war than in the treatment of fractured limbs. In these cases the comfort of the patient as well as the restoration of the limb to a normal condition depends very largely upon the apparatus used in treatment; indeed, there are not a few instances where even the life of a patient may be saved by the employment of appliances which permit of adequate surgical and nursing attention, without interference with the position of the injured parts.

Fig. 1.—Showing Splint and Pulley Attachments.

Miss McCaul has endeavoured to interest the Army Medical Service of this country, as well as civilian surgeons, in a hammock suspension device for fractures, which is being extensively used by the French Government with most beneficial results. The invention is the result of the genius of an American artist, Miss Gassette, who has lived many years in Paris and who has spent much time and pains during the war in devising various aids for the injured by well-calculated arrangements of spring and balance. Briefly, the principle of the appliance in question may be described in two words, "traction" and "suspension." The principle as applied to fractured legs is by no means original; it entered into appliances employed both by English and French surgeons long before the war. The special feature, however, of Miss Gassette's adaptation of the principle is that her apparatus seems to permit of greater facility than any other in the dressing of the wounds without interference with the immobility of the bone fragments.

The apparatus is really a double-extension splint, which provides a hammock for the fractured leg, which is suspended from a frame, adaptable to any bed, in such a way that accurate counter-balance is obtained. The method of securing extension will perhaps more easily be understood by a comparison of this splint with a Hodgen splint; in the latter extension is secured by the pull of gravity against the fixing at the foot attachment. In the former case extension is obtained by weights swung from pulleys attached to each end of the limb. Provision is made for unlacing any part of the hammock when dressing a wound without disturbing the position. The arrangement secures the comfort of the patient, for he can turn in any position without endangering the immobility of the limb.

Fig. 2.—Showing Method of Arranging Transverse Bar, with Pulley Attachments, on the Chassis (or Gallows).

A similar appliance is made for putting up an arm, and attachments are provided for dealing with a dropped foot or dropped wrist.

## Figures and Tables

**Fig. 1. f1:**
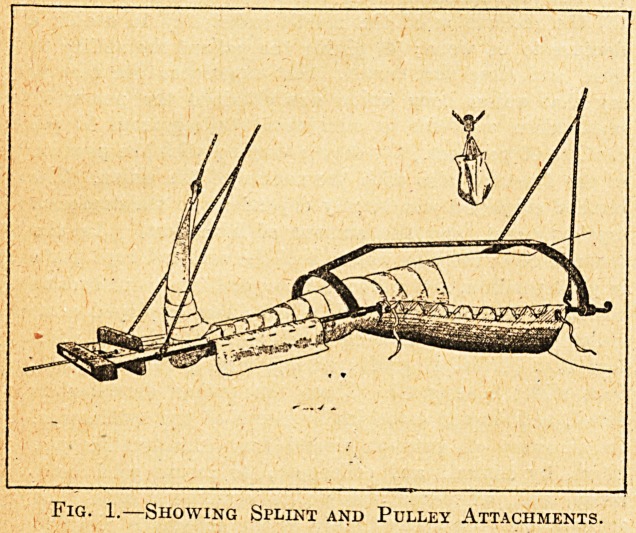


**Fig. 2. f2:**